# Weight patterns and perceptions among female university students of Karachi: a cross sectional study

**DOI:** 10.1186/1471-2458-13-230

**Published:** 2013-03-16

**Authors:** Zubaida Sirang, Hassaan Hasan Bashir, Bilal Jalil, Sarah Haroon Khan, Samia Altaf Hussain, Aneeqa Baig, Maryam Taufeeq, Kashif Samad, Muhammad Masood Kadir

**Affiliations:** 1 Medical College, Aga Khan University, Karachi, Pakistan; 2 Professor, Department of Community Health Sciences, Aga Khan University, Karachi, Pakistan

## Abstract

**Background:**

Body weight and its perception play an important role in the physical and mental well-being of a person. Weight perception is found to be a better predictor of weight management behaviour as compared to actual weight. In Pakistan, studies have been done on the prevalence of weight status but weight perception is still unexplored. The study was done to examine relationships between body weight perception, actual weight status, and weight control behaviour among the female university students of Karachi.

**Methods:**

A cross sectional study was carried out during Sep-Nov 2009 on female students in four universities of Karachi, Pakistan. Our final sample size included 338 female university students. Height and weight were measured on calibrated scales. A modified BMI criterion for Asian populations was used.

**Results:**

Based on measured BMI; the prevalence of underweight, normal weight and overweight females was 27.2%, 51.5% and 21.3% respectively. As a whole, just over one third (33.73%) of the sample misclassified their weight status. Among underweight (n=92), 45.70% thought they were of normal weight. No one who was truly underweight perceived them self as overweight. Among the normal weight (n= 174), 9.8% thought they were underweight and 23.6% considered themselves overweight. Among the overweight (n=72); 18.3% considered themselves normal. Only one female student thought she was underweight despite being truly overweight.

**Conclusions:**

Our study shows that among female university students in Karachi, the prevalence of being underweight is comparatively high. There is a significant misperception of weight, with one third of students misclassifying themselves. Underweight females are likely to perceive themselves as normal and be most satisfied with their weight. Health policy makers should implement these findings in future development of health interventions and prevention of depression, social anxiety and eating disorders associated with incorrect weight perception among young females. Studies that employ a longitudinal approach are needed to validate our findings.

## Background

Body weight and its perception is an important aspect of health and constitute a significant role in physical and mental well-being. The World Health Organization (WHO) uses the body mass index (BMI) to categorize weight status. The BMI is calculated as the weight in kilograms divided by the square of the height in meters (kg/m2). A BMI <18.5 is considered underweight and >25 overweight. Falling between these ranges is normal weight. Based on this definition, the WHO estimates more than one billion adults to be overweight and three hundred million of them to be obese [1].Obesity is a well-known risk factor for cardiovascular diseases [2], diabetes [3] and decreased life expectancy [4]. Much less investigated and even less discussed is being underweight. Underweight people are at risk for irregular menstruation [5], weak immunity [6] and osteoporosis [7].

Current prevalence of different body weights show varying trends in different parts of the world. The majority of these studies are from the developed world. A study of 2032 high school male and female students across the U.S., grades 9 through 12, found 1.5% of students to be underweight and 51.2% and 47.4% to be normal weight and overweight respectively [8]. Another study, focused on 360 first year female university students in South Africa found 7.2% to be underweight and 10% overweight [9].

In Pakistan, few studies are available to ascertain the prevalence of weight status. One study of 284 schoolchildren in Karachi found that 52% were underweight [10]. The results are strikingly different from the studies elsewhere although effects of age-group have to be kept in mind. In Pakistan, socio-economic level plays a major role in weight status and is a focus of the study done previously [11]. No studies of weight perception can be found despite the fact that it influences eating habits and other aspects of mental health in Pakistan [12].

Regardless of whether a person is underweight, normal or overweight, weight perception is an important determinant of nutritional habits and weight management [10]. Discrepancies between true weight and weight perception can have serious implications. Normal or underweight adolescents, who perceive themselves as overweight, are at risk for eating disorders such as anorexia nervosa [13].

Conversely, overweight adolescents perceiving otherwise are unlikely to control diet or engage in physical activity [14]. Aberrant weight perceptions can lead to risky behaviour [15] such as smoking cigarettes and skipping breakfast. Underweight women are more likely to have poorer psychological health than normal weight women. In contrast, overweight and obese women are more likely to have poor health related behaviours and lack of internal locus of control compared with normal weight women [16]. The psychosocial, psychological and behavioural implications are significant.

Females are sensitive to the effects of weight perception [17] and studies have shown that they perceive weight incorrectly [18-21]. The problems with weight and weight perception are universal but greatly influenced by social norms. Current studies are heavily concentrated in the developed world. More widespread studies are needed to properly assess the importance of the patterns and perceptions of weight status. There is a lack of information from the South Asian population. Preventive strategies, dealing with the psychological risk at an individual level and body norms in media can be addressed. It is important to study health behaviour of young adults because the rapidly changing conditions in the world are bringing about changes in behaviour, and behaviour formed in the second decade of life has lasting implications for the individual in particular and the public health in general [22]. This study was done to explore the relationships between body weight perceptions, actual body mass index and weight control behaviours among the female students of Karachi, Pakistan.

## Methods

### Study population

A cross sectional study was carried out from September to October 2009 on female university students in the city of Karachi, Pakistan. Eight well-recognized universities in different areas of Karachi were approached, of which four refused participation. Each of the remaining four universities was dedicated to one field of study; medicine, the arts, business and engineering.

The sample, 400 female students, was equally divided between the four institutions to make it as representative of the female student population as possible. After obtaining written consent, students were requested to fill out a questionnaire and proceed to a private area to have their height and weight measured. Although we approached 100 students at each institution, the final completed questionnaires in hand were 88, 100, 96 and 99 respectively from the four institutions. The loss of questionnaires was due to various reasons including students refusing to continue mid-way, leaving the institution unannounced and misplacing questionnaires. Hence, our overall response rate was 95.8%.

Students with a diagnosed eating disorder, depression, heart disease or diabetes were excluded from the analysis. We also chose to exclude questionnaires in which measurements of height and weight were not available. As a result of these criteria, 7 students were excluded due to missing height and weight measurements and 38 due to the diagnosis of the chronic conditions listed above.

The final sample size included 338 female university students.

### Questionnaire

The questionnaire for this study included 35 items divided into five sections. It contained questions about demographic characteristics, self-reported measurements, weight satisfaction, weight-changing practices, physical activity levels, body shape concerns and reasons for weight concern. Physical activity and body shape concern was assessed using validated tools (described later).

### Measures

#### Actual weight status

Height and weight were measured after completion of the questionnaires by two separate investigators. Height was measured to the nearest 0.1 cm using a set square and standard calibrated scale against a wall. Weight was measured to the nearest 0.5 kg using a calibrated digital scale. Measurements were collected with participants in either thin socks or barefoot and with heavy clothing items removed. Body mass index (BMI) was computed as weight in kilograms divided by the square of height in meters and categorized according to the World Health Organization modified Asian criterion [23]. This sets normal BMI as 18.5 to 23.0, with anything below or above this range as underweight and overweight respectively.

### Weight perception

Participants were asked to describe their body weight in terms of the following responses: underweight, slightly underweight, normal, slightly overweight, and overweight. These five responses were collapsed into three; underweight, normal and overweight in the analysis. This item was used to reflect self-perception of body weight status. They were not asked about their perceived height and the ideal weight according to the height.

### Weight-changing practices

Items in the questionnaire asked participants what they were trying to do about their perceived weight; lose, gain, maintain or nothing at all. The amount of weight change desired was asked. Similar items questioned weight-related practices and methods employed to modify weight.

### Body shape

Female body shape is the cumulative product of a woman’s skeletal structure and the quantity and distribution of muscle and fat on the body.

To measure concerns with body shape, a shortened 8-item version of the Body Shape Questionnaire (BSQ) developed by Evans et al. was used [24].

### Physical activity

The last 7 day, short form of the International Physical Activity Questionnaire (IPAQ) was used which was developed as an instrument for cross-national monitoring of physical activity and inactivity [25].

Specific criteria were used to categorize participants into low, moderate or high physical activity levels according to IPAQ instructions.

### Ethical statement

The study was granted approval by the Ethics Review Committee of the Aga Khan University, Karachi. Informed written consent was obtained through a consent form that was given to the participants along with the questionnaire.

### Statistical methods

The data from the questionnaires was entered using Epi-data 3.1. Data set was exported to SPSS v.15 for complete analysis. Statistical analysis was carried out for the complete sample as well as for three different groups which were created according to measured BMI: underweight, normal and overweight. Mean values and standard deviation for all continuous variables: weight, height, BMI and BSQ score for all groups were obtained. Frequencies for each categorical variable were calculated for each group as well. To determine the differences regarding each categorical variable in each BMI group,chi sq test was done. The difference between the weight status and perceived weight was statistically significant on application of Chi-square test.

## Results

The mean age of the 338 participants enrolled in the study was 20.64 ± 1.49 years (Table 1).

**Table 1:** Weight status according to BMI and according to perception of the female university students

**Variables**	**N**	**Percentage (%)**
Mean Age	20.64 ± 1.49	
Weight status according to BMI		
Under -weight	92	27.2
Normal weight	174	51.5
Over weight	72	21.3
Weight status according to perception		
Under- weight	68	20.1
Normal weight	171	50.59
Over weight	99	29.28

### Actual weight status

The mean weight, height and BMI of the participants were found to be 53.81 kg (± 9.78), 1.61 m (±0.06) and 20.89 (±3.74) respectively. Based on measured BMI; the prevalence of underweight, normal weight and overweight females was 27.2%, 51.5% and 21.3% respectively (Table 1).

### Perceptions of weight status

As a whole, over one third (33.7%) of the sample misclassified their weight status (Table 2). Among females who were actually underweight (n=92), 54.3% perceived their weight status correctly and 45.7% thought they were of normal weight. No one who was underweight perceived them self as overweight. Among the normal weight (n=174), 66.7% perceived their weight status correctly, 9.8% thought they were underweight and 23.6% considered themselves overweight. Overweight female students (n=72) were found to have more accurate judgment; 80.3% correctly perceived themselves overweight whereas 18.3% considered themselves normal. Only one female student thought she was underweight despite being overweight.

**Table 2:** Comparison of actual weight with perceived weight exhibiting significant levels of misclassification among female students

	**Underweight n=92**	**Normal weight n=174**	**Over weight n=72**	***P*-value**
**Mean BMI (SD)**	17.23 (1.07)	20.5 (1.26)	26.45 (3.47)	
**Weight Perception**				
Underweight	54.3% (50)	9.8% (17)	1.4% (1)	
Normal weight	45.7% (42)	66.7% (116)	18.3% (13)	p <0.001
Over weight	0	23.6% (41)	80.3% (58)	
**Misclassified cases as% of total sample**	12.43% (42)	17.16% (58)	4.14% (14)	

### Weight related perceptions and practices

Underweight, normal weight and overweight students differed significantly with regard to perceptions of weight, weight goals and weight loss practices. These differences are shown in Table 3. Underweight females, being most satisfied with their weight (70.7%), were mostly doing nothing about their weight (62.2%). One fifth (20%) were trying to gain weight and a very small percentage (4.4%) was trying to lose. They also had the least negativity towards themselves about their weight (6.5%). Just above half (56.7%) skipped breakfast but not many were actively avoiding certain foods because of their weight (14.4%).

**Table 3:** Weight satisfaction, dietary habits, weight goals and self-negativity among different weight groups in female students

	**Underweight n=92**	**Normal weight n=174**	**Overweight n=72**
Mean BMI (SD)	17.23 (1.07)	20.53 (1.26)	26.45 (3.47)
**Weight Satisfaction**			
Satisfied	70.7% (65)	57.5% (100)	20.8% (15)
Not satisfied	22.8% (21)	33.9% (59)	68.1% (49)
Not sure	6.5% (6)	8.6% (15)	11.1% (8)
**Dietary habits**	(n = 90)	(n=171)	(n=70)
Skipping breakfast	56.7% (51)	55.6% (95)	62.9% (44)
Not skipping	43.3% (39)	44.4% (76)	37.1% (26)
Avoiding foods	14.4% (13)	44.4% (76)	71.4% (50)
**Not avoiding**	85.6% (77)	55.6%(95)	28.6% (20)
**Weight Practices**	(n=90)	(n=171)	(n=69)
Trying to lose	4.4% (4)	25.1% (43)	49.3% (34)
Maintaining	13.3% (12)	24.6% (42)	23.2% (16)
Trying to gain	20.0% (18)	2.9% (5)	0
Doing nothing	62.2% (56)	47.4% (81)	27.5% (19)
**Self-negativity**	(n=92)	(n=169)	(n=71)
Yes	6.5% (6)	16.6% (28)	40.8% (29)
No	78.3% (72)	72.8% (123)	40.8% (29)
Cant say	15.2% (14)	10.7% (18)	18.3% (13)

Females who were at normal weight shared similar low negativity towards themselves (16.6%) but also lower satisfaction with weight (57.5%). A quarter (25.1%) was still trying to lose weight. Almost half were not trying to change their weight at all which corresponds to their weight category. Very few (2.9%) were still trying to gain. Approximately half of the normal category were both skipping breakfast and avoiding certain foods (55.6% and 44.4%).

Overweight females, having the most accurate perception of weight, were the least satisfied with their weight (20.8%) although negativity was equally present and absent (40.8%). Overweight females skipped breakfast the most (62.9%) and avoided foods the most (71.4%). Just under half were trying to lose weight (49.3%) with 27.5% doing nothing about their weight. No overweight female was trying to gain weight. Table 4 compares the ‘perceived’ weight groups on the basis of weight goals. The weight goals according to perceived categories were very similar to the weight goals of the measured categories despite the fact that one third of females misperceived their weight.

**Table 4:** Weight goals according to perceived weight category among female students of Karachi Pakistan

		***Perceived weight***		
	**Underweight (n=66)**	**Normal weight (n=168)**	**Overweight (95)**	***P-value***
**Weight goals**				
Trying to gain	22.7% (15)	4.8% (8)	0	p < 0.001
Trying to lose	3.0% (2)	20.2% (34)	47.4% (45)	
Maintaining	9.1% (6)	27.4% (46)	18.9% (18)	
Doing nothing	65.2% (43)	47.6% (80)	33.7% (32)	

### Body shape

A mean score of 75.34 was obtained on the BSQ with a standard deviation of 38.6. The majority (83.7%) scored ‘low’ (<112), 4.5% scored ‘medium’ (112–128) and 11.8% scored ‘high’ (>128). The lowest mean scores were obtained in the underweight category (55.25 ± 20.11) and the highest in the overweight category (101.32 ± 46.03) as shown in Table 5.

**Table 5:** Mean BSQ score (SD) and physical activity levels of three weight groups among female students

	**Underweight n=92**	**Normal weight n=174**	**Overweight n=72**	***P*-value**
**Mean BSQ Score (SD)**	55.25 (20.11)	75.2 (36.45)	101.32 (46.03)	
**Physical Activity Level**				
Low	63.04% (58)	54.60% (95)	51.39% (37)	p < 0.001
Moderate	30.43% (28)	29.89% (52)	22.22% (16)	
High	6.52% (6)	15.52% (27)	26.39% (19)	

### Physical activity

According to IPQA specific criteria majority of students, 58.28%, were classified as ‘low’ physical activity levels.

Physical activity levels were found to be mostly low in all categories relative to BMI as well. 63.04% of underweight females reported low physical activity levels, as did 54.6% and 51.39% of normal and overweight females respectively. The highest percentage of females with high physical activity levels (26.39%) was observed in the overweight category.

## Discussion

Our socio-demographics revealed that our study population was mostly unmarried, healthy female students. Our mean BMI of 20.89 ± 1.49 falls in line with other studies performed on similar target populations in Pakistan [26,27]. Based on this result, it can be asserted that although we had a convenience sampling, the similarity in mean BMI with other studies counters the presence of any strong bias in our results. The breakup of our sample into weight categories according to the WHO Asian criterion showed that just more than half of our sample was of normal weight with an approximately equal proportion being underweight and overweight; underweight being slightly higher. This distribution is similar to the one reported in other studies performed on university students in Pakistan [27,28] but is strikingly different to results from studies in the west. The proportion of underweight females in similar target populations from the west is much lower and varies from 3 - 18% [8,9,16]. The proportion of underweight female students in our study (27.2%) is similar to other studies from Pakistan [27,28] but higher to other Asian countries such as India [29] and Lebanon [30], where the underweight females were found to be 12.1% and 12.4% respectively. Studies from Pakistan that comment on the underweight status of young female adults are scarce. Regarding proportions of overweight, our results (21.3%) fall in line with studies from other countries such as America, South Africa and Swedan [8,9,16] in which overweight ranges from approximately 10 - 25%. This suggests that the difference in prevalence of overweight status from east to west is much less as compared to the difference in underweight status. In all studies, the normal weight status follows similar trends in proportion.

The higher proportion of underweight status is of concern as it put females at risk of many health issues i.e. menstrual irregularitites, osteoporosis and weak immunity [5-7]. Previous research on weight perception has shown that female students do not have a realistic perception of their actual weight [18-21]. One third of our study population misclassified their weight which mirrors this assertion and has been shown in other studies [20]. Approximately half of the underweight females in our study perceived themselves as normal which implies that underweight students may not want to gain weight but would prefer to remain underweight. The same concept applies to the normal weight students who perceive themselves as overweight as they may attempt to unnecessarily reduce weight to meet their inaccurate normal standards [31]. The tendency of underweight females to perceive themselves as normal is frequently observed [8] but the emphasis usually shifts to normal weight females perceiving themselves as overweight. These normal weight females may undertake unnecessary weight loss which is a more apparent issue than the risks of remaining underweight. The more significant finding was the perception of normal weight by underweight females which may have its roots in a wider social and cultural acceptance of thinness.

Our results show that the normal weight females had a fair level of accuracy in weight perception. We found that overweight females had the most accurate perception of weight and this has been shown to be the case in other studies too [32]. Our results indicate that the highest weight satisfaction was seen in the underweight group. This is potentially dangerous as these female students are less motivated to normalize their weight, which was seen in the high percentage of them doing nothing about their weight. Reassuringly, very few underweight females were actively trying to lose weight but on the other hand. This shows the poor weight related perceptions in the underweight group. A fairly large part of the normal and overweight groups were not satisfied with their weights which have also been reported for female students in the UK [33] and undergraduates in the US [34]. The high level of dissatisfaction among the normal group is worrisome as a quarter of them still want to lose weight. The high level of dissatisfaction and active weight loss practices among the overweight group are justified. However, it is important to emphasize realistic weight loss goals and acceptable weight loss practices. Skipping breakfast and actively avoiding foods was seen across the board with a tendency to increase with BMI. These unhealthy practices have been associated with greater levels of fatigue [35], increased weight gain [36], and irregular menstrual cycles [37].

A unique finding in our study is the assessment of weight goals in the perceived weight categories. The results are very similar to the weight goals of the measured weight categories. This indicates that although many studies report weight perception as a better indicator of weight management behaviour [8,9], it seems that this is not applicable to our population. It appears from the results that despite weight perception being inaccurate overall, the weight goals did not differ significantly between perceived and measured weight groups. Self-perception of weight did not supersede true weight in influencing weight goals. Further studies should be done to evaluate this trend and to determine the possible other factors, such as socio-cultural pressures or lack of weight-related knowledge, that may be responsible for poor weight goals. Comparing the mean BSQ score of our study (75.34) with others (85.0 to 96.3) [38] we encountered a slightly lower value, portraying the students being less concerned about their body shape overall. Correlating weight subgroups with the mean BSQ scores however, we did find an increase in mean BSQ scores from underweight to overweight students. This is consistent with other studies which show a positive association between mean BSQ scores and BMI [39]. Thus, body shape does concern female students in our population but not as much as other studies have shown.

Trends in physical activity as highlighted by previous studies [38] reveal persons with normal BMI engaging in more exercise than the underweight or overweight groups. We observed a different trend, with low physical activity being the majority in all three weight groups. High levels of physical activity were reported most frequently from the overweight group, where weight dissatisfaction was high and the majority was trying to lose weight. Regardless of the physical activity levels in each subgroup, the majority of our entire sample reported “low” physical activity levels. This indicates a poor prevalence of physical activity among female university students in the city which should be a cause for concern.

### Study limitations

It was a cross sectional study on female university students of Pakistan. Gaining admission to university must involve some selection for socioeconomic status (which affects nutrition), more urban upbringing, and perhaps better health. The findings of our study may not be generalized to Pakistani women of the same age.

We had a convenient sampling of both institutions and students. To reduce the bias introduced, we tried to represent female university students as accurately as possible by choosing students of different disciplines and exclude students who may not be in control of the quality of their diet and exercise level (those with chronic diseases). Additionally, voluntary participation in a study that involves anthropometric assessment could have caused a bias towards students who do not have weight issues. We also could not account for students who chose not to answer our additional questions or who reported their responses incorrectly.

Also, the reasons for the different weight perception among the participants were not assessed.

## Conclusions

Our study has shown that among female university students in Karachi, the prevalence of being underweight is comparatively high. There is a significant misperception of weight, with one third of students misclassifying themselves. Despite the misperception of weight, weight goals were not seen to be very different among measured and perceived weight groups. Health policy makers should implement these findings in future development of health interventions and prevention of depression, social anxiety, low self-esteem and eating disorders among young females due to incorrect perception of weight. If large populations such as university students learned healthy behaviours early in life, this would not only have favourable health outcomes at an individual level, it would also reduce the burden on health services.

Large scale longitudinal studies should be performed in Pakistan dealing with weight perception and specific aspects of dietary habits, body shape and physical activity.

## Competing interests

The authors declare that they have no competing interests.

## Authors’ contributions

ZS contributed to the study design, performed statistical analysis and wrote the manuscript.HHB contributed to the study design, performed the statistical analysis, drafted the manuscript. BJ contributed to the study design, performed the statistical analysis and drafted the manuscript. SH participated in study recruitment and design. SAH participated in study recruitment and design. AB participated in study recruitment and design. MT participated in study recruitment and design. KS participated in study recruitment and design. MK participated in planning and designing the study and edited the manuscript. All authors read and approved the final manuscript.

## Pre-publication history

The pre-publication history for this paper can be accessed here:

http://www.biomedcentral.com/1471-2458/13/230/prepub
